# Interaction of Musicianship and Aging: A Comparison of Cortical Auditory Evoked Potentials

**DOI:** 10.1155/2015/545917

**Published:** 2015-10-04

**Authors:** Jennifer L. O'Brien, Dee A. Nikjeh, Jennifer J. Lister

**Affiliations:** ^1^Department of Psychology, University of South Florida St. Petersburg, 140 7th Street S, DAV 116, St. Petersburg, FL 33701, USA; ^2^Department of Communication Sciences and Disorders, University of South Florida Tampa, 4202 E. Fowler Avenue, Tampa, FL 33621, USA

## Abstract

*Objective*. The goal of this study was to begin to explore whether the beneficial auditory neural effects of early music training persist throughout life and influence age-related changes in neurophysiological processing of sound. *Design*. Cortical auditory evoked potentials (CAEPs) elicited by harmonic tone complexes were examined, including P1-N1-P2, mismatch negativity (MMN), and P3a. *Study Sample*. Data from older adult musicians (*n* = 8) and nonmusicians (*n* = 8) (ages 55–70 years) were compared to previous data from young adult musicians (*n* = 40) and nonmusicians (*n* = 20) (ages 18–33 years). *Results*. P1-N1-P2 amplitudes and latencies did not differ between older adult musicians and nonmusicians; however, MMN and P3a latencies for harmonic tone deviances were earlier for older musicians than older nonmusicians. Comparisons of P1-N1-P2, MMN, and P3a components between older and young adult musicians and nonmusicians suggest that P1 and P2 latencies are significantly affected by age, but not musicianship, while MMN and P3a appear to be more sensitive to effects of musicianship than aging. *Conclusions*. Findings support beneficial influences of musicianship on central auditory function and suggest a positive interaction between aging and musicianship on the auditory neural system.

## 1. Introduction

The perception of music infuses the human brain with a rich auditory sensory experience and is a cognitively complex task requiring the integration of multiple cortical levels and neural systems. Because of the intense training and skill acquisition a musician receives from an early age, a musician's brain provides unique opportunities to explore the impact of music perception and music training on neural structural and functional adaptation and development.

Ground-breaking anatomic studies using functional magnetic resonance imaging (fMRI) [1, 2] revealed that musicians compared to nonmusicians have 5% greater cerebellar volume, significantly larger anterior corpus callosum, and increased gray matter volume in the left Heschl's gyrus and left inferior frontal gyrus. In addition to altered cortical structure, electroencephalographic (EEG) and magnetoencephalographic (MEG) recordings of cortical auditory evoked potentials (CAEPs) parallel anatomic studies showing cortical enlargement of auditory areas important for music perception [3, 4] and reveal superior preattentive auditory sensory memory representations for musicians compared to nonmusicians [5–14]. Specifically, findings suggest that musicians relative to nonmusicians (a) discriminate auditory differences that are undetectable for nonmusicians at a preattentive processing level implying that sensory memory traces containing auditory information may be enhanced by musical expertise [8], (b) discriminate embedded pitch shifts faster (shorter latencies) within familiar and unfamiliar interval patterns [5], (c) have larger amplitude responses to changes in relative pitch structure, such as melodic contour and interval [6], rhythmic deviation [15], and timbre of expertise instrument [16], (d) respond faster (shorter latencies) to pitch deviances of harmonic tone complexes [9, 12], and (e) enhanced sensitivity to acoustic changes of harmonic complexes and speech syllables [10, 13, 14]. Interestingly, effects of musicianship on the plasticity of CAEPs can be seen even after short-term (e.g., two weeks) musical training (e.g., [17]).

These remarkable effects of music training on auditory neural structure and function have been observed from childhood through young adulthood; however, research is limited when it comes to exploring whether these auditory neural effects of music training extend throughout the life of the musician. Given their history of extraordinary exposure to and experience with spectrally and temporally rich auditory stimuli at an early age, it is questioned whether older adult musicians might experience age-related changes in central auditory processing differently than older nonmusicians.

It is a well-known fact that older adults often complain that although they can hear speech, they cannot understand it. Age-related physiological changes typically alter the way in which spectral and temporal information are encoded [18]. Older adults often exhibit neural activation patterns that are qualitatively different and more frontally oriented than those of younger adults [19–21]. These patterns suggest relatively slower neural transit time and altered auditory inhibition/arousal by irrelevant stimuli for older adults and characterize an inefficient aging auditory system that contributes to poor speech understanding in noisy, real-world listening environments [21]. Given that young adult musicians have altered auditory neural structures and superior auditory neural processing for music and speech, is it then possible that older adult musicians may have a more efficient auditory system than their nonmusician cohorts?

Investigations of age-related changes affecting auditory neural processing have used both behavioral and electrophysiological measures (e.g., EEG, MEG). Psychoacoustic measures provide information about the listener's perception of sound and reflect a conscious attentional process. Recent behavioral studies exploring the rate of age-related decline on peripheral and central auditory processing between older adult musicians and age-comparable nonmusicians (ages 45–65) reported that older musicians demonstrate increased auditory working memory [22] and less age-related decline for gap-detection and speech-in-noise thresholds than nonmusicians [20]. Findings suggest that musical training may diminish the impact of age-related auditory decline [22] and, further, that musicians may experience less age-related decline in central auditory processing than nonmusicians [23]. We question what impact, if any, the interaction between musicianship and aging has on preattentive neurophysiological processing of sound for older adults.

Preattentive cortical auditory evoked responses (e.g., P1-N1-P2, MMN, and P3a), the focus of the current study, are particularly suitable when investigating responses of older adults because they reflect the automatic detection and discrimination of acoustic sensory memories prior to attentional focus without being contaminated by attention, motivation, or cognitive demands of the task [24, 25]. CAEPs provide excellent temporal resolution and allow for a noninvasive evaluation of the various stages of auditory processing from preattentive sensory perception to later cognitive levels. To date, CAEP studies comparing older musicians and nonmusicians are unknown.

CAEP studies investigating age-related changes of central auditory processes in older nonmusician listeners have reported similar aging effects on P1-N1-P2 elicited by pure tones [19] and gap-detection [21]. The P1-N1-P2 complex reflects the physiological detection of audible stimulus energy [28]. In both studies comparing older and younger adults with normal hearing, there was a pattern of larger P1 amplitudes and slower P2 latencies for the older adults. For gap-detection, P1 latency was earlier for older adults than younger; however, there was no P1 latency difference between older and younger adults for detection of pure tones. In neither study did N1 or P2 amplitudes or the N1 latency differ by age group. It may be speculated that larger P1 amplitude and earlier P1 latency as well as longer P2 latencies may represent an adaptive neural response to aging. That is to say, compared to younger adults, older adults may activate or recruit more or different neural resources to increase the efficiency of stimulus detection and inhibition and may have slower processing or extended neural conduction time once the auditory stimulus is detected.

Mismatch negativity (MNN) is a passively elicited negative potential typically considered to be independent of attention and higher cognitive processing [29]. In general, the MMN is thought to be a preperceptual or preattentive central auditory response to an acoustic deviation based on the detection of an auditory regularity in a preceding sound sequence and an automatic sensory memory-based comparison process [30, 31]. Indexing in sensory memory as reflected by the MMN is considered to be a preattentive because it has been shown to occur in the absence of attention and even conscious awareness [30]. Note that, while the MMN can be elicited outside of attention, it can also be influenced by attentional modulation (see [47] for modeling evidence of temporal attention enhancing the MMN).

While the MMN can occur preattentively, the P3a, which often follows the MMN, is associated with an involuntary and automatic shift of attention to and conscious perception of a deviant or new stimulus [30, 32, 33]. Typically, the P3a is elicited in response to infrequent task-irrelevant stimuli that are unexpected (e.g., pitch “C”) in a sequence of frequently presented standard stimuli (e.g., pitch “A”) within which the listener is actively attending to the detection of infrequent target stimuli (pitch “B”). The infrequent presence of pitch “C” automatically shifts the listener's attention to that tone, resulting in a P3a.

Age-related studies using CAEPs to elicit MMN and P3a are limited; however, results are consistent. Comparisons of older and younger adults suggest that MMN is affected more by stimulus contrast and presentation rate than age alone [19, 26, 34]. Age alone did not affect the MMN latency or amplitude for stimulus-change detection of frequency [19] or stimulus duration with a short interstimulus interval (ISI = 0.5 sec); rather, as the time between the stimuli increased, the MMN attenuated more for older listeners than younger suggesting that MMN may reflect the gradual decay of the stimulus trace in the auditory system and that this trace decays faster for older than younger adults [26, 34]. We are aware of only two studies of P3a and aging for auditory stimuli. Those studies indicate that P3a is absent [27] or delayed [35] for older as compared to younger adults, indicating that older adults are slower to switch attention to and evaluate distracting auditory stimulus outside of their focused attention.

Our current research sought to examine cortical auditory evoked responses of older musicians and is based on the framework of our earlier research with young adult musicians [9, 10]. It was an exploratory study to investigate the interaction between the aging process and possible effects of early music training and the possible impact of this interaction on the neurophysiological processing of sound. Preattentive CAEPs elicited by harmonic tones are compared between normal hearing older musicians and their nonmusician cohorts. In addition, CAEP data from older musicians and nonmusicians is compared to that of their younger counterparts [9] to further elucidate effects of age-related changes on central auditory processing. Specifically, the P1-N1-P2 complex was elicited by a standard harmonic tone stimulus to establish the physiological detection of sound at the level of the auditory cortex. MMN and P3a were elicited by small deviant frequencies (i.e., 1.5% and 6% lower in frequency than a standard) of a harmonic tone complex. Based on previous research of the P1-N1-P2 potentials [10, 19, 21] and the assumption that larger P1 amplitude reflects increased neural recruitment for regulating incoming auditory stimuli in older adults, it was predicted that, given their musical expertise and training, older musicians may have smaller P1 amplitudes for harmonic tones than older nonmusicians and similar group latencies. We also predicted a shorter P2 latency for older musicians compared to older nonmusicians, given the general increase in P2 latency with age and reduction effects of musicianship on aging.

Based on previous electrophysiological research among young adult musicians and nonmusicians, it was predicted that older musicians may have shorter MMN and P3a latencies and larger MMN amplitudes than age-comparable nonmusicians for harmonic complexes; however, no significant effects of musicianship were predicted for P3a amplitudes. MMN and P3a amplitude were predicted to increase as the size of frequency deviance increased. Based on the literature described above, the following aging effects were predicted for P1-N1-P2, MMN, and P3a: (1) P1 amplitude would be larger; (2) P1 latency is earlier and P2 latency later for older adults compared to younger; (3) neither P2, N1, or MMN amplitudes nor MMN latency would be affected by age; and (4) P3a amplitude would be smaller and latency later for older compared to younger adults.

## 2. Method

### 2.1. Participants

Eight older adult musicians (mean age = 62.5 yrs, range = 58–67 yrs) and eight older nonmusicians (mean = 61.4 yrs, range = 55–70 yrs) were recruited from local communities, community music organizations, and the University of South Florida (USF). Musicians averaged 11.5 years of formal music training that began between the ages of 8 and 16 (see Table 1 for musical history). Nonmusicians had fewer than 12 months of music training. For the purpose of this investigation, formal music training refers to a minimum of 6 years of professionally directed and implemented music instruction and technical exercises provided by a professional musician and/or music educator [36]. All participants were right-handed and native speakers of English and had normal pure-tone thresholds (less than or equal to 25 dB HL bilaterally) for the frequencies of interest in the present study (≤3000 Hz). Performance on the Words-in-Noise (WIN) [37, 38] test, in terms of the dB signal-to-noise ratio for 50% correct word recognition in noise, was age-appropriate for all participants. A summary of hearing thresholds and WIN scores, shown in Table 2, indicates that the hearing sensitivity of the two older adult groups was highly similar. None had a history of neurological impairment, absolute pitch ability, exposure to tone languages, or previous participation in psychoacoustic experiments. The study was approved by the USF Institutional Review Board and documented informed consent was obtained from all subjects. Participants were reimbursed $15 per hour of participation.

The identical inclusion and exclusion criteria were used for our earlier recruitment of 40 young adult musicians and 20 nonmusicians [9]. Young adult musicians were between the ages of 18 and 33 (mean age = 22 years) and had an average of 9.8 years (median = 9.18 yrs, range = 5 to 17 yrs) of formal music training. Nonmusicians were between the ages of 20 and 34 (mean age = 23) and had fewer than 12 months of music training.

### 2.2. Stimuli

Harmonic complexes were digitally generated (sampling rate = 50 kHz), controlled, and presented using a Tucker-Davis Technologies (TDT) RP2 Real-Time Processor with model HB 7 headphone buffer. Duration of all stimuli was 200 ms and shaped with a cos^2^ window to create a 10 ms rise/fall time. Interstimulus interval (ISI) was 500 ms. All stimuli were presented bilaterally via Etymotic Research (ER2) insert earphones at 75 dB SPL.

The present study is a continuation of a series of studies investigating the relationship between pitch perception and vocal production by types of musicians (i.e., vocalists, string instrumentalists, and wind instrumentalists) and nonmusicians [10]. Thus, auditory stimuli consisted of harmonic tone complexes containing fundamental frequencies (*F*0) that occur within the mid-frequency range of the female vocal register, *F*0 = 261.63 Hz to 392 Hz (C4 to G4) [39]. The standard tone was G4, *F*0 = 392 Hz, and was chosen from a behavioral task in a previous study because this tone elicited the best overall difference limen for frequency (DLF) across groups [10]. The two deviant harmonic complexes, *F*0 = 386 Hz (HMD1.5), 1.5% difference from the standard tone *F*0 (an eighth tone difference) and *F*0 = 370 Hz (HMD6), 6% difference from the standard tone *F*0 (a semitone difference), were selected based on DLFs measured behaviorally in a companion study and represented a range of behavioral performance. In physical terms, the interval between adjacent whole tones is a 12% difference between the fundamental frequencies of each tone. The interval between a reference tone and its semitones is a fundamental frequency difference of roughly 6% (e.g., G4 to *F*4# equals 392 Hz to 370 Hz). Each harmonic tone stimulus contained a *F*0 and three harmonics. The amplitude of each harmonic was divided by its harmonic number to create a natural amplitude contour in the frequency spectrum.

### 2.3. Procedure

#### 2.3.1. Electroencephalographic Recording

CAEPs were recorded and analyzed using a Compumedics Neuroscan EEG system with a SynAmps 2 amplifier and Neuroscan Scan 4.3 acquisition software. A cap of 64 sintered electrodes was placed on the subject's head with additional electrodes above and below the left eye and at the outer canthus of each eye to monitor eyeblink activity. The nose served as reference and the forehead served as ground. To minimize any auditory attentive behavior, the participant was comfortably seated in a sound-attenuated booth and instructed to watch a closed-caption movie of choice and to ignore the auditory stimuli. All impedances were kept below 30 kΩ. The acquisition of EEG data was by continuous sampling, recorded at an AD (analog to digital) sampling rate of 1000 Hz and stored on the computer for offline averaging. The raw signal was amplified within a frequency band of 0.05–100 Hz.

The harmonic tone condition was designed as a multideviant oddball paradigm to elicit MMN and P3a. The protocol consisted of one standard tone (*F*0 = 392 Hz) and two infrequently occurring deviant tones (HMD6 and HMD1.5). The standard tone occurred on a minimum of 75% (1080 minimum) of the trials and the two deviant frequencies occurred on 25% (180 per deviant, 360 total deviants) of the trials. Stimuli were presented in a pseudorandom sequence with at least three standard stimuli separating presentations of deviant stimuli; thus, two deviant stimuli did not occur in succession. The response to the standard stimulus was analyzed to establish physiological detection of the auditory stimuli (P1-N1-P2).

#### 2.3.2. Data Analysis

Offline analysis of the continuous EEG waveforms was conducted using Neuroscan Scan 4.3 Edit software and began with manual artifact rejection. As a precaution for data analysis, the first 10 CAEP responses were omitted from the averaging process to exclude the variation of the N1 amplitude (i.e., the refractoriness) associated with the start of the stimulation sequence [40, 41]. EEG epochs of 700 ms (−100 to 600 ms) were obtained, baseline corrected (−100–0 ms), and averaged separately for the standard and deviant stimuli. To eliminate ocular movement contamination, epochs containing artifacts exceeding ±80 *μ*V in the horizontal and vertical eye channels were rejected from averaging. CAEP waves were digitally band-pass filtered at 0.1–30 Hz with a squared Butterworth zero-phase filter (12 dB/octave roll-off). In order to maximize signal-to-noise ratio at Fz, all of the processed average files for MMN/P3a analysis were individually rereferenced to the mastoids.

P1-N1-P2 response had the largest amplitudes measured at Fz; thus, reported measures and statistical analysis are based on CAEP responses measured from the Fz electrode for the standard stimulus. P1 was identified as the first positive peak occurring between 25 and 90 ms in the group average CAEP waveforms [17]. The N1 was defined as the largest negativity occurring between 70 and 140 ms. The P2 was defined as the largest positivity occurring between 140 and 255 ms. Latency windows of ±25 ms around each amplitude peak for each group were determined and individual peak amplitudes and latencies were quantified within these preselected windows using scripts within the Neuroscan Scan 4.3 Edit software; the script selected the time point with the highest (for P1 and P2) or lowest (for N1) amplitude value within the designated window. Visual inspection of individual waveforms was conducted to ensure that clear peaks did not fall outside of the designated windows for any participant.

The MMN is illustrated by a difference wave obtained by subtracting the averaged CAEP elicited by the standard stimulus from the averaged CAEP elicited by a deviant stimulus [42]. Difference waveforms to illustrate MMN and P3a were calculated for each deviant stimulus condition. The MMN response was largest at electrodes Fz and Cz with the largest amplitudes measured at Fz. Thus, reported measures and statistical analysis are based on CAEP responses measured from Fz. The MMN was verified by polarity inversion at the mastoids prior to rereferencing all individual files to the mastoids. MMN has been shown to invert in polarity at electrodes below the level of the Sylvian fissure [42]. Polarity inversion at the mastoids is an accepted method to verify the MMN response to tonal changes [41]. CAEP amplitudes were quantified by first determining the peak latencies from the grand-average difference waves separately for each deviant as the largest peak between 100 and 300 ms at Fz for MMN [43]. The P3a was chosen as the first positive peak following the MMN.

Grand average group difference waveforms for each harmonic tone deviant stimulus were derived from the Fz electrode for purposes of illustrating MMN and P3a and for selecting latency windows. Latency windows of ±25 ms around each amplitude peak for each group and deviant condition were determined [44]. MMN and P3a peak amplitudes and latencies for each participant were quantified within these preselected windows for each harmonic deviant condition using scripts within the Neuroscan Scan 4.3 Edit software to select the time point with the highest (for P3a) or lowest (for MMN) amplitude value within the designated window. Visual inspection of individual waveforms was conducted to ensure that clear peaks did not fall outside of the designated windows for any participant.

## 3. Results

Peak amplitude and latency data were analyzed using IBM SPSS Statistics software (version 21.0). To determine the effect of age and musicianship on P1-N1-P2, MMN, and P3a, univariate analyses of variance (ANOVAs) were conducted on individual mean component latencies and amplitudes. An alpha level of 0.05 was used for all statistical tests.

### 3.1. Effects of Musicianship within Older Adults

#### 3.1.1. P1-N1-P2 Components

**Figure 1:**
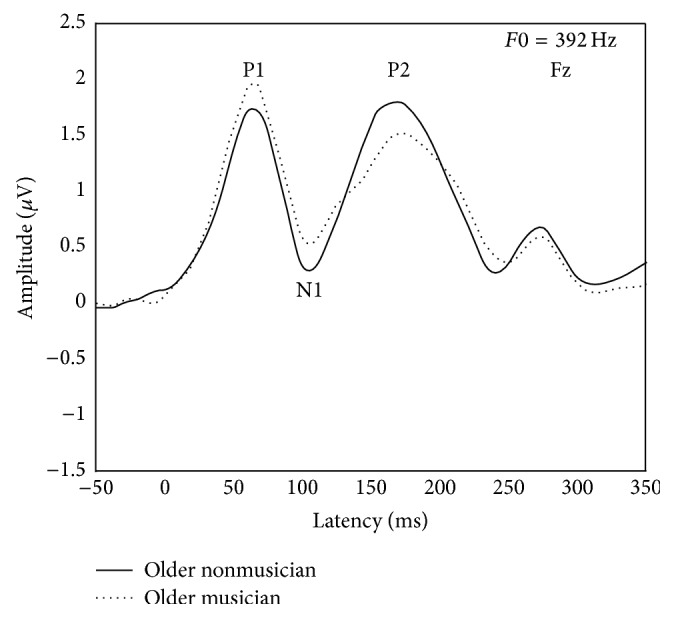
Group averaged CAEP waveforms illustrating P1, N1, and P2 elicited from older nonmusicians (solid line) and older musicians (dotted line) by the standard harmonic tone (*F*0 = 392 Hz).

Analysis of P1-N1-P2 was conducted at Fz for the standard harmonic tone stimulus (*F*0 = 392 Hz). The peaks are designated in Figure 1 for visual inspection and the latency and amplitude means and standard deviations are reported in Table 3. There were no significant group differences of P1, N1, and P2 latencies and amplitudes between older adult musicians and nonmusicians, *F*(1,14) = 1.19, *p* = 0.293 for P1 latency; *F*(1,14) = 0.18, *p* = 0.676 for P1 amplitude; *F*(1,14) = 0.71, *p* = 0.412 for N1 latency; *F*(1,14) = 0.34, *p* = 0.567 for N1 amplitude; *F*(1,14) = 0.11, *p* = 0.742 for P2 latency; and *F*(1,14) = 0.23, *p* = 0.638 for P2 amplitude.

#### 3.1.2. Mismatch Negativity (MMN)

**Figure 2:**
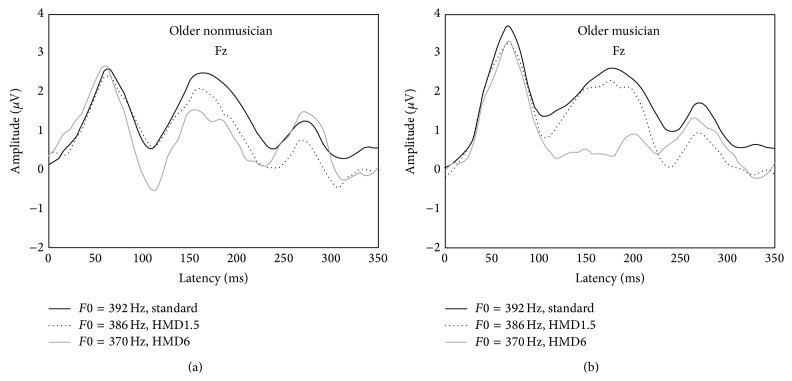
Grand average group CAEP waveforms at Fz for older nonmusicians (a) and older musicians (b). A solid black line represents the CAEP to the standard stimulus while the dotted and gray lines represent CAEPs to deviants presented in the oddball paradigm (i.e., HMD1.5 and HMD6).

**Figure 3:**
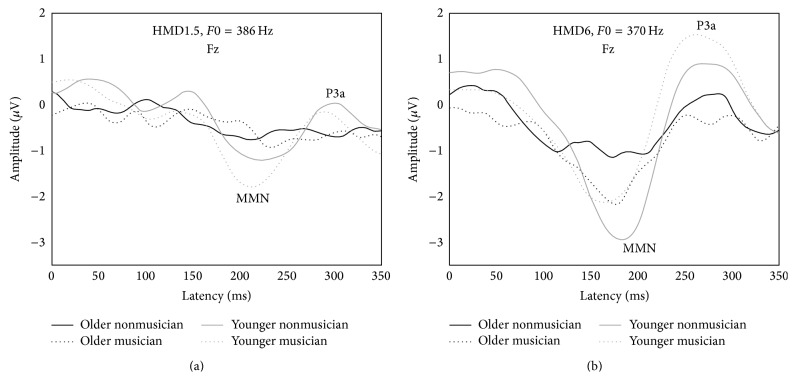
Grand average group difference waveforms (deviant minus standard) at Fz from older adult musicians (OM), older nonmusicians (ONM), young musicians (YM), and young nonmusicians (YNM) in response to a 1.5% frequency deviance (HMD1.5, (a)) and a 6% frequency deviance (HMD6, (b)).

The MMN and P3a were elicited from older musicians and nonmusicians by two harmonic tone pitch deviants and are illustrated by comparing CAEPs to the standard stimulus and CAEPs to the deviant stimuli elicited from the oddball paradigm (Figure 2). The MMN and P3a latency and amplitude means and standard deviations are reported in Table 4. The grand average group CAEP difference waveforms reflect the MMN and P3a for the two frequency deviances (Figure 3). Parallel two-way repeated-measures ANOVAs were conducted on individual mean MMN and P3a latencies and amplitudes for each stimulus condition with the within-subject factor Stimulus (HMD1.5 [*F*0 = 386 Hz], HMD6 [*F*0 = 370 Hz]) and the between-subject factor Musicianship (older nonmusicians, older musicians). MMN latencies to harmonic tone pitch deviants were significantly shorter for older adult musicians than older nonmusicians, *F*(1,14) = 7.96, *p* < 0.001. Further, there was a significant effect of deviant magnitude, *F*(1,14) = 200.68, *p* < 0.001 for MMN latency. Across groups, as the magnitude of the frequency deviance increased, response latency decreased (Figure 3; see Table 4 for mean group latency values). The interaction between Musicianship and Stimulus was also significant, *F*(1,14) = 7.57, *p* = 0.016 for MMN latency. A post hoc analysis of the significant interaction revealed that the effect of Stimulus on MMN latency was significant for both older nonmusicians (*p* < 0.001) and older musicians (*p* < 0.001). The effect of Musicianship on MMN latency was also significant for both HMD1.5 (*p* < 0.001) and HMD6 (*p* = 0.005).

In contrast, MMN amplitude did not differ significantly by Musicianship, *F*(1,14) = 1.66, *p* = 0.218; however, there was a positive effect of deviant magnitude, *F*(1,14) = 5.35, *p* = 0.036. For all, as the magnitude of the frequency deviance increased, response amplitude increased. Interaction between Musicianship and Stimulus was not significant, *F*(1,14) = 1.04, *p* = 0.326 for MMN amplitude.

#### 3.1.3. P3a Component

Overall, P3a latency occurred significantly earlier for older adult musicians than older nonmusicians, *F*(1,14) = 12.65, *p* = 0.003 (Figure 3). For all participants, the larger frequency deviance elicited a shorter latency response, *F*(1,14) = 114.90, *p* < 0.001 (Table 4). There was no interaction between group membership and the magnitude of the frequency deviant, *F*(1,14) = 2.64, *p* = 0.126. P3a amplitude was not dependent on Musicianship, *F*(1,14) = 0.59, *p* = 0.454, nor magnitude of deviance, *F*(1,14) = 3.07, *p* = 0.102. The interaction was not significant, *F*(1,14) = 0.50, *p* = 0.492.

### 3.2. Effects of Age and Musicianship

Amplitude and latency of CAEPs P1-N1-P2, MMN, and P3a, elicited from older adult musicians and nonmusicians by harmonic tone complexes, were compared to CAEPs previously elicited from young adult musicians and nonmusicians [9] in univariate ANOVAs with Age (younger, older) and Musicianship (musicians, nonmusicians) as the between-subject factors. CAEP data from older and younger musicians is compared to further examine and elucidate the effects of age-related changes on central auditory processing. All of the previously collected data from young adult musicians and nonmusicians was reanalyzed using methods described in this paper.

#### 3.2.1. P1-N1-P2 Components

**Figure 4:**
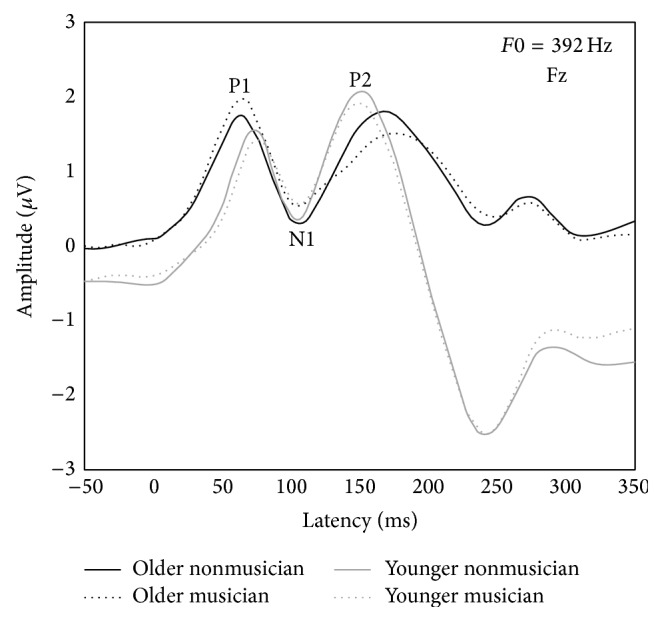
Group averaged CAEP waveforms from older (black lines) and young (gray lines) adult nonmusicians (solid lines) and musicians (dotted lines) illustrating P1-N1-P2 at Fz elicited by the standard harmonic tone (*F*0 = 392 Hz).

P1-N1-P2 latency and amplitude means and standard deviations for all four groups are reported in Table 3. P1 latencies were significantly affected by Age, *F*(1,72) = 14.93, *p* < 0.0011, with earlier latencies for older adults than younger listeners, but was not affected by Musicianship, *F* < 1. There were no significant effects of Age or Musicianship or interaction between the two for P1 amplitude, N1 latency, or N1 amplitude, *F*s < 1. P2 latencies were significantly affected by Age, *F*(1,72) = 64.75, *p* < 0.001, with significantly earlier latencies for younger listeners compared to older listeners, but was not affected by Musicianship or the interaction of Age and Musicianship, *F*s < 1. P2 amplitude was not affected by Age, *F*(1,72) = 2.44, *p* = 0.112, or Musicianship or the interaction of the two, *F*s < 1. Averaged group waveforms for P1-N1-P2 components are illustrated in Figure 4.

#### 3.2.2. Mismatch Negativity (MMN)

MMN latency and amplitude means and standard deviations are reported in Table 4. There were significant effects of Age for MMN latency: younger participants had shorter latencies than older participants for HMD1.5, *F*(1,72) = 155.81, *p* < 0.001, and HMD6, *F*(1,72) = 10.81, *p* = 0.002. Younger participants also had significantly larger MMN amplitudes than older participants for HMD1.5, *F*(1,72) = 6.21, *p* = 0.015, and for HMD6 *F*(1,72) = 5.43, *p* = 0.023. There were also significant effects of Musicianship for MMN latency: musicians had shorter latencies than nonmusicians for HMD1.5, *F*(1,72) = 60.75, *p* < 0.001, and HMD6, *F*(1,72) = 33.74, *p* < 0.001. Effects of Musicianship on MMN amplitude for both stimuli were not significant, *Fs* < 1.

MMN latency effects for HMD1.5 were qualified by an Age × Musicianship interaction, *F*(1,72) = 42.44, *p* < 0.001. The two young adult groups (YM, YNM) had very similar, short latencies; the OMs had slightly longer latencies and the ONMs had the longest latencies of all. The latencies of the YM and YNM groups were not significantly different, *F*(1,72) = 1.77, *p* = 0.187, but all other groups showed latencies significantly different from each other, *p*s < 0.001. MMN amplitude effects for HMD6 were qualified by an Age × Musicianship interaction, *F*(1,72) = 4.45, *p* = 0.038. The largest amplitude overall was found for the YNM group while the amplitudes of the two musician groups (YM, OM) were very similar and smaller than that of the YNM. The ONM group showed the smallest overall amplitude. The difference between the YMs and OMs was not significant, *F* < 1, but YNMs had significantly larger amplitudes than ONMs, *F*(1,72) = 9.15, *p* = 0.003.

#### 3.2.3. P3a Component

P3a latency and amplitude means and standard deviations are reported in Table 4. There were significant effects of Age on P3a latency for HMD1.5, *F*(1,72) = 55.00, *p* < 0.001, and on P3a amplitude for HMD6, *F*(1,72) = 8.90, *p* = 0.004. There were also significant effects of Musicianship for P3a latency: musicians had shorter latencies than nonmusicians for HMD1.5, *F*(1,72) = 11.52, *p* = 0.001, and HMD6, *F*(1,72) = 21.06, *p* < 0.001. Effects of Musicianship on P3a amplitude for both stimuli were not significant, *F*s < 1. P3a latency effects for HMD6 were qualified by an Age × Musicianship interaction, *F*(1,72) = 9.21, *p* = 0.003. P3a latency for HMD6 was shortest for the OM group, followed closely by the YM group, and longest latencies were observed for the YNM and ONM groups, respectively. P3a latency for the YM group was not significantly different from that of the OM or YNM groups but all other group comparisons were significant.

## 4. Discussion

Musicians from early childhood through young adulthood have been shown to have enhanced auditory sensory-memory and sensitivity to acoustic changes for music and speech; however, there is a paucity of research exploring whether older musicians retain this acoustic processing advantage throughout life. This was an exploratory study using preattentive CAEPs, specifically the P1-N1-P2, MMN, and P3a components, to compare responses between formally trained older musicians and an age-comparable group of nonmusicians. Moreover, to investigate the interaction between aging and musicianship, CAEPs from the older musicians and nonmusicians in the current study were compared to CAEPs of young adult musicians and nonmusicians from an earlier study [9]. As an exploratory study, the sample size for the older adult groups was small (*n*'s = 8) compared to the younger adult group previously published (musicians *n* = 40; nonmusicians *n* = 20) [9, 10]; however, the data are useful in showing neurophysiological responses of musicianship that are age-agnostic as well as responses that are age-specific.

Consistent with our hypotheses and similar to our findings among young adults, preattentive neurophysiological responses to music stimuli do distinguish older adult musicians from older nonmusicians. Unique to this investigation is the added comparison of preattentive CAEPs between groups of young and older musicians and nonmusicians and findings that further differentiate the obligatory evoked potentials from the change-detection potentials. It appears that the early obligatory P1-N1-P2 complex may be more affected by aging than musicianship, whereas the inverse is true of the preattentive responses representing the change-detection paradigm. That is, MMN and P3a derived components appear to be influenced by musicianship rather than aging.

### 4.1. P1-N1-P2 Complex

As predicted, older adults regardless of music background detected the presence of acoustic energy earlier than younger adults (i.e., shorter P1 latency); yet once arousal was triggered, neural conduction was longer from P1 to P2 (i.e., later P2 latency) for older adults than the younger. Findings are consistent with others [19, 21] and suggest perhaps an age-related adaptive neural response that increases neural recruitment to initiate auditory arousal to the presence of acoustic energy. Once aroused, the delayed time-course may be explained by age-related refractory-time differences between younger and older slowing of synchronous neural firing in the central auditory pathways. That is, once the neurons are activated, the older system takes longer to recover before the neurons can fire again. This increase in refractory time results in slower neural travel time.

We expected an effect of both aging and musicianship on P1 amplitude and found neither. We also expected an effect of musicianship on P2 latency in older adults, which was not present in this data set. As for the effect of aging on P1 amplitude, it is well documented that older listeners typically demonstrate an increase in the strength of the obligatory response (P1 amplitude) as a result of an adaptive increase in neural recruitment for the physiological detection of the auditory stimulus [18, 19, 21]. Thus, it was predicted that, as a group, the older listeners would have larger P1 amplitude than the young listeners. As for the influence of musicianship, smaller P1 amplitude has been shown to reflect reduced processing demands [45]. Therefore, it was speculated that highly trained listeners, such as musicians, would be more efficient at inhibiting irrelevant stimuli and regulating familiar stimuli (i.e., harmonic tones) than untrained listeners and, thus, musicians were predicted to have smaller P1 amplitude than nonmusicians. One plausible explanation for the absence of significant differences may be the choice of stimulus. The P1-N1-P2 complex was elicited by a single repetitive harmonic tone. A review of aging studies indicates that both the latency and amplitude patterns of P1, N1, and P2 may be altered depending on the level and spectrum of the stimulus as well as the presentation paradigm (for review see [46]). It is plausible that an adaptive increase in neural activation (i.e., larger P1 amplitude) may have been unnecessary to perceive this less complex auditory stimulus; whereas, previously reported P1 amplitude findings have been linked to more complex stimuli such as neural detection of gaps in noise [21], pure tones with and without noise [19], and detection of consonant-vowel speech syllables [25]. Another possible explanation may be due to the small number of older participants compared to the larger group of young participants. Future research will have larger numbers of older participants more compatible in size to the younger groups.

### 4.2. MMN and P3a Components

MMN and P3a derived components appear to be more sensitive to neural effects of musicianship than aging. Consistent with our hypotheses, MMN and P3a elicited by small deviant frequencies in harmonic tones distinguished older adult musicians from older nonmusicians. Prior to attentional focus, older musicians discriminated small changes of pitch in harmonic tone complexes faster (i.e., shorter MMN latencies) than older untrained listeners indicating superior sensory memory-based comparison processes and supporting the premise that early and intensive music training may affect central auditory processing throughout life. Consistent with the literature, for both groups of older listeners, as the magnitude of stimulus deviance increased, MMN latency decreased and amplitude increased [5, 9, 24]. As predicted, MMN amplitude did not significantly differ between the two older groups suggesting that older musicians discriminated changes in pitch faster than nonmusicians without an increase in neural activation.

P3a latency and amplitude patterns for the older adult groups were similar to the MMN, but not identical. Older musicians switched attention faster (i.e., shorter P3a latency) to acoustic changes in music without an increase in neural activation. This difference between trained and untrained listeners speaks to the musician's extensive auditory experience and exposure to multiple, concurrent, and sequential music stimuli. Consequently, even a minor change in pitch elicited a swift shift of attention toward the distraction. Like the MMN latency, all older listeners responded faster to the larger frequency deviance (earlier P3a latency).

To further elucidate the interaction between aging and musicianship, MMN and P3a data were compared among four groups: young musicians (YM), young nonmusicians (YNM), older musicians (OM), and older nonmusicians (ONM). For the larger frequency deviance (i.e., 6% or 22 Hz), musicianship appears to have the advantage; that is, all the musicians (YM and OM) reacted to the pitch change faster with comparable neural effort than the nonmusicians (YNM and ONM). Since attentional modulation of the MMN is possible [47], this effect of musical expertise on the MMN could in part be due to a difference in the attentional demand of the stimuli since tone stimuli might be inherently more “interesting” for musicians, or musicians might be more used to directing their attention to the sounds.

The YNM had the strongest neural activation (largest MMN amplitude), while the ONM had the smallest. Findings support previous literature suggesting that preattentive change-detection (MMN) may be affected more by stimulus contrast and presentation rate than age alone [19, 26, 34]. However, despite the fact that the older adults had normal pure-tone thresholds up to 3 kHz and that low-frequency stimulus contrasts were used to minimize the confounding effects of age-related high-frequency hearing loss, the possibility remains that physiological discrimination of the 1.5% pitch deviance (6 Hz difference) may have been compromised by aging effects.

P3a latency patterns were similar to those of the MMN. Like the MMN response to the larger harmonic tone deviant, musicianship appears to have prevailed. All musicians (YM and OM) compared to the untrained listeners (YNM and ONM) demonstrated faster preattentive registration of a deviant harmonic stimulus outside attentional focus and shifted their attention toward the distraction. This reflexive auditory-neural response may be a residual adaptation related to music training and experience during childhood. Consistent with the MMN response to the smaller 1.5% pitch deviance, older adults were slower to register this distraction outside of their attentional focus than the younger adults and neural effort was not a distinguishing factor. In summary, MMN and P3a components appear to be more influenced by musicianship rather than aging. Further, it appears that early and intensive music training may alter aging effects on central auditory processing throughout life.

## 5. Conclusions

Within the neuroscience of music, this study was an initial foray into the investigation of the effects of aging and musicianship on the auditory neural system. Youth and musicianship appear to be an advantageous combination for efficient and enhanced preattentive auditory neural processing. In terms of physiological detection of sound, P1-N1-P2 appears to be more sensitive to effects of aging rather than musicianship. All older listeners, regardless of musicianship, demonstrated faster auditory arousal to the presence of acoustic energy than all younger listeners suggesting an adaptive increase in neural recruitment; yet once arousal was initiated, neural conduction of acoustic energy was slower for the older adults than younger. In terms of automatic acoustic discrimination, MMN and P3a appear to be more affected by musicianship than aging. All musicians, regardless of age group, demonstrated a pattern of enhanced auditory sensory-memory-based comparison processes for harmonic tone stimuli and exceptional sensitivity to and involuntary distraction by an acoustic change of music. Findings suggest that musicianship has beneficial neurophysiological consequences on central auditory processing throughout life, and, further, some of these neurophysiological effects may be independent of age-related changes.

The lifelong ability to adapt to environmental demands and sensory stimulation is based on the dynamic capacity of the human brain to modify and alter its structure and function. Formal music training has been shown to facilitate and enhance encoding of the acoustic signal, shape subcortical, and early cortical stages of acoustic perception and may retard age-related neural changes and facilitate adaptive neural function. The benefits of music training are socially, clinically, and educationally relevant. Music training and practice may be used as an educational tool or a rehabilitative strategy to facilitate neurophysiological processing of sound. The possibilities for off-setting age-related physiological changes of the auditory neural system through music training have far-reaching effects not only for the field of neuroscience and music education, but also for gerontology, speech-language pathology, and hearing science. Regardless of the remarkable technological advances in hearing devices such as programmable digital hearing aids and cochlear implants, none can duplicate our original auditory system. Consequently, it is essential to investigate other means by which our auditory abilities may be enhanced and protected.

This was an introductory study to explore possibilities that early music training may influence auditory processing in later life and to assess the methodological challenges faced when working with an older population. Implications from this study have been interpreted with caution and generalizations were kept to a minimum due to the small sample of older musicians and the intrasubject variability that occurs when working with older adults. Future recommendations for studies of older adults include recruiting larger numbers to offset individual variability and allowing for frequent breaks during the experiment to offset participant fatigue. To further elucidate the effects of aging and musicianship, it would be beneficial to include higher-level listening tasks using more complex auditory stimuli such as speech stimuli and to also include psychoacoustic measures of auditory perception. Finally, while the information presented suggests that formal music training and extensive auditory sensory exposure facilitate neurophysiological responses throughout life, the influence of genetic factors and inherent musical ability cannot be dismissed. It is yet unknown whether musical abilities and cortical structural differences are due to learning or whether these differences reflect innate abilities and capacities that are advanced by early exposure to music.

## Figures and Tables

**Table 1:** Summary of older musician musical history.

Musician	Age (years)	Age training initiated (years)	Instrument/voice	Total years training	Total years music making	Average hours per week
OM1	61	8	I	10	52	4
OM2	67	8	I	7	59	2
OM3	61	8	I/V	20	29	2
OM4	58	10	I/V	8	20	0
OM5	65	13	I/V	17	52	13
OM6	64	8	I	8	50	2
OM7	59	16	I/V	8	43	3
OM8	65	8	I/V	14	57	8
Mean	**62.5 yrs**	**9.87 yrs**		**11.5 yrs**	**45.25 yrs**	**4 h 15 mins/wk**
Range	**58–67 yrs**	**8–13 yrs**		**7–20 yrs**	**20–59 yrs**	**0–13 hrs**

**Table 2:** Mean pure-tone hearing thresholds (dB HL) and 50% signal-to-noise ratios (dB) for the Words-in-Noise (WIN; [38, 39]) test plus standard deviations for each older participant group.

	WIN	Ear	Frequency (Hz)							
			250	500	1000	2000	3000	4000	6000	8000
Older nonmusicians (*n* = 8)	5.3 (2.4)	Right	15 (5)	14 (5)	15 (6)	16 (7)	16 (3)	26 (17)	26 (9)	30 (22)
		Left	13 (3)	16 (5)	13 (7)	16 (6)	22 (6)	26 (15)	29 (12)	26 (13)

Older musicians (*n* = 8)	5.6 (2.7)	Right	18 (6)	14 (5)	14 (8)	14 (13)	16 (12)	16 (8)	28 (11)	31 (23)
		Left	13 (3)	16 (6)	15 (8)	14 (11)	13 (9)	19 (9)	26 (11)	31 (17)

**Table 3:** Mean and standard deviation values for P1-N1-P2 cortical auditory evoked potential latencies and amplitudes elicited by standard harmonic tone (*F*0 = 392 Hz) from older and younger musicians and nonmusicians.

	P1		N1		P2	
	Latency (ms)	Amplitude (microvolts)	Latency (ms)	Amplitude (microvolts)	Latency (ms)	Amplitude (microvolts)
Older nonmusicians	65 (8)	2.02 (1.1)	104 (10)	−0.02 (0.95)	171 (15)	2.0 (1.4)
Older musicians	61 (8)	2.24 (0.8)	109 (12)	0.27 (1.1)	174 (15)	1.69 (1.0)
Younger musicians	74 (10)	1.84 (0.75)	105 (11)	0.21 (0.88)	147 (10)	2.21 (1.1)
Younger nonmusicians	74 (10)	2.00 (0.76)	103 (11)	0.199 (1.2)	146 (11)	2.43 (0.81)

**Table 4:** Mean and standard deviation values for MMN and P3a cortical auditory evoked potential latencies and amplitudes elicited by deviant harmonic tones (HMD 1.5 and HMD 6) from older and younger musicians and nonmusicians.

		MMN		P3a	
		Latency (ms)	Amplitude (microvolts)	Latency (ms)	Amplitude (microvolts)
Older nonmusicians	HMD 1.5	298 (15)	−1.1 (1.3)	330 (20)	−0.06 (1.3)
	HMD 6	200 (19)	−1.62 (0.9)	283 (13)	0.81 (1.1)

Older musicians	HMD 1.5	238 (12)	−1.26 (0.5)	314 (18)	−0.18 (0.8)
	HMD 6	172 (15)	−2.6 (1.7)	251 (16)	0.19 (1.5)

Younger nonmusicians	HMD 1.5	219 (18)	−1.90 (1.1)	297 (14)	0.43 (1.5)
	HMD 6	183 (12)	−3.44 (1.4)	265 (16)	1.47 (1.4)

Younger musicians	HMD 1.5	213 (14)	−2.21 (1.3)	284 (14)	0.20 (1.3)
	HMD 6	163 (15)	−2.63 (1.5)	259 (15)	2.04 (1.6)

## Abbreviations

ANOVA: Analysis of variance
CAEPs: Cortical auditory evoked potentials
DLF: Difference limen for frequency
EEG: Electroencephalographic
*F*0: Fundamental frequency
fMRI: Functional magnetic resonance imaging
HMD1.5: Stimulus with 1.5% difference from the standard tone *F*0
HMD6: Stimulus with 6% difference from the standard tone *F*0
ISI: Interstimulus interval
MEG: Magnetoencephalographic
MMN: Mismatch negativity
ONM: Older nonmusicians
OM: Older musicians
TDT: Tucker-Davis Technologies
USF: University of South Florida
WIN: Words-in-Noise
YNM: Younger nonmusicians
YM: Younger musician.
